# Air pollution, respiratory illness and behavioral adaptation: Evidence from South Korea

**DOI:** 10.1371/journal.pone.0221098

**Published:** 2019-08-13

**Authors:** Tackseung Jun, In-sik Min

**Affiliations:** 1 Department of Economics, Kyung Hee University, Seoul, South Korea; 2 Department of Economics, Barnard College, Columbia University, New York, New York, United States of America; Louisiana State University System, UNITED STATES

## Abstract

Air pollution is closely associated with the development of respiratory illness. Behavioral adaptations of people to air pollution may influence its impact, yet this has not been investigated in the literature. Our hypothesis is that people experience and learn the underlying air quality to decide their adaptation, and they have a stronger incentive to behaviorally adapt to the air quality as it deteriorates. We tested our hypothesis on a sample of approximately 25,700 individuals from South Korea from 2002 to 2013 that contained information on daily doctor’s visits due to respiratory disease. We matched individuals to the mean of the past seven-day concentration of the particulate matter of size between 2.5 and 10 micrometers (PM_10_) in their county of residence. We examined whether people living in counties with greater air pollution suffer less from respiratory disease when the concentration increases. For the analysis, we separated counties into quintiles based on their mean seven-day PM_10_, and regressed the binary indicator of a daily doctor’s visit with a resulting diagnosis of respiratory disease on the seven-day PM_10_ concentration of the county of residence interacted with the quintile dummies. The key findings are that a 1-standard-deviation increase in the seven-day PM_10_ concentration in the two lowest quintiles is associated with an increase of 0.054 percentage points in the likelihood of a doctor’s visit with a resulting diagnosis of respiratory disease, which is about 40% larger than the effect in higher quintiles, and the size of 1-standard-deviation gradually increases from 0.037 percentage points in the third quintile to 0.040 percentage points in the fifth quintile. The smaller increase in the likelihood of respiratory disease in more polluted locations can be explained by the behavioral adaptation to the environment, but the effectiveness of the adaptation seems limited among the highly polluted locations.

## 1. Introduction

Air pollution has significant adverse health effects on people. In 2013, 5.5 million premature deaths worldwide, or 1 in every 10 total deaths, were attributable to air pollution. These deaths cost the global economy about $225 billion USD in labor income lost [1]. Air pollution is especially severe in some of the world’s fastest-growing urban regions in low and middle income countries. This raises further concern that people may myopically endure the depreciation of air quality as the price to pay for more income, while dirty air can have serious long-term consequences [2–4].

The science is clear in linking breathing polluted air to the deterioration of respiratory function and the subsequent development of respiratory illness. Particles deposited in the respiratory tract in sufficient amounts can induce inflammation, and airway inflammation increases airway responsiveness to irritants and may reduce lung function [5–10]. Much empirical evidence supports the adverse impact of air pollution. As an example, living close to streets with a high traffic density is found to be a risk factor for the occurrence of respiratory disease [11–17]. A meta-analysis of birth cohorts found a clear association between air pollution and respiratory infections, such as pneumonia [18]. However, the degree to which respiratory illness is influenced by air pollution differs greatly among these studies. Some studies reported no significant relationship between exposure to polluted air and the occurrence of respiratory disease [19–22].

Here, we hypothesize that (i) people experience and learn the underlying air quality to adapt themselves to reduce the adverse health effects of air pollution, and (ii) they have a stronger incentive for behavioral adaptation as the air quality deteriorates. According to this hypothesis, the impact of polluted air on the likelihood of respiratory disease should be modulated by human behaviors. Studies in this line of research [23–27] found a lower hospital admission rate due to respiratory illness on days with active pollution alert. This finding is consistent with the hypothesis that people attempted to avoid exposure to pollution.

The behavioral response to the daily pollution alert is only one part of the greater underlying behavioral adaptations to living with air pollution. Many other behavioral adaptations may require structural changes in one’s life. For example, people build indoor facilities for outdoor activities, install air-purification system in buildings, and enforce law to reduce emissions from vehicles and factories, and so on. Therefore, behavioral adaptations to polluted air are numerous and diversified, yet many of them are unobserved, which makes encompassing all the dimensions of behavioral adaptation infeasible.

How do we assess the impact of diverse adaptations on respiratory disease, without listing all possible behavioral adaptations? Our empirical strategy is based on the observation that many of these behavioral adaptations stem from one’s learning and experience of the environment. A classic example is a behavioral adaptation to local climate conditions. The studies [28–30] found that mortality from extremely hot temperature is smaller in regions with more frequent hot temperature events, suggesting that people adapted themselves to local temperature conditions, for example by installing air-conditioning systems.

In this paper, we assume that people are accustomed to the underlying air quality of the place they live in and base their adaptation decision on it. As long as the factors that could influence the air quality of the county remain stable, the concentration of a major pollutant in a county could represent the underlying air quality of the county, and can be a proxy for the degree of behavioral adaptation of the people in the county. Our regression model tests whether people who live in a place of higher concentration are better adapted to pollution and thus suffer less from respiratory disease due to an increase in the ambient pollutant concentration.

## 2. Materials and methods

### 2.1 Data processing and description

The data on individual patients was based on an extraction from the National Health Insurance System (NHIS) of South Korea. We randomly extracted 30,000 individuals who existed in the database of the NHIS at January 1st, 2002, and kept track of them until December 31st, 2013. For each person, we extracted gender, age category, county of residence, and the daily information on doctor’s visits with the classification of diagnosed diseases according to the Korea Classification of Disease (KCD-6). The information on doctor’s visits is used to construct a daily-level binary indicator of a doctor’s visit with a resulting diagnosis of respiratory disease, which is classified as codes J00 to J99 and R00 to R09 according to the KCD-6 (The details of disease for each code are listed in S1 Table). Acute respiratory infections in the upper and lower tracts, which are closely related to ambient pollution [31, 32], explain about 68% of the diagnoses of respiratory disease (S1 Table).

The daily mean concentration of particulate matter of size between 2.5 and 10 micrometers (PM_10_) at station level between January 1st, 2002 and December 31st, 2013 was used to represent the degree of air pollution. A network of 333 air-monitoring stations throughout South Korea collects hourly samples of PM_10_. These pollutants are inhalable particles and small enough to penetrate the thoracic region of the respiratory system, potentially affecting the likelihood of respiratory disease [33, 34].

We matched the individual data with the seven-day PM_10_ concentration, if available, of the county the individual lived in. If the county had more than one station, then the mean of the PM_10_ concentrations from the stations was assigned to individuals in the county. People living in counties with no air monitoring system were excluded from the sample.

Moreover, the factors that may influence exposure to air pollution should be taken into account in the regression to estimate the effect of the PM_10_ concentration on respiratory disease. For example, comfortable outdoor temperature will favor outdoor activities, which will increase exposure to ambient pollution. In this paper, the station-level daily maximum temperatures, obtained from weather-monitoring stations operated by the Korea National Weather Service, are used to construct a county-level daily maximum temperature series, and to define the comfortable temperature ranges (maximum temperature between 20°C and 26°C) for outdoor activities.

The resulting sample for the analysis, after matching and merging data on patients, ambient pollution and temperature, runs from January 1st 2002 to December 31st 2013 and has about 25,700 individuals per year from 236 counties (S2 Table). The changes in population over the sample periods are due to changes in the availability of the pollutant information, and a natural attrition of the sample as people decease. The population of ages 1–9 shrinks over time as people in this age category get older to move into the higher age group, but no one is newly recruited to the sample. Approximately 50.1% of population are women. People of ages 20–39 have the largest share (36.2%) in the sample, and the oldest (ages 60–89) have the lowest (10.7%) (S2 Table).

Regarding the frequency of respiratory disease, approximately 0.57% of observations have had a doctor’s visit with a resulting diagnosis of respiratory disease (S3 Table). This can be interpreted as the average likelihood of respiratory disease. The likelihood is the highest among people of ages 10–19, with 1.6%, and the lowest among people of ages 20–39, with 0.42%. Females (0.64%) have a greater chance of respiratory disease diagnosis than males (0.49%).

**Fig 1:**
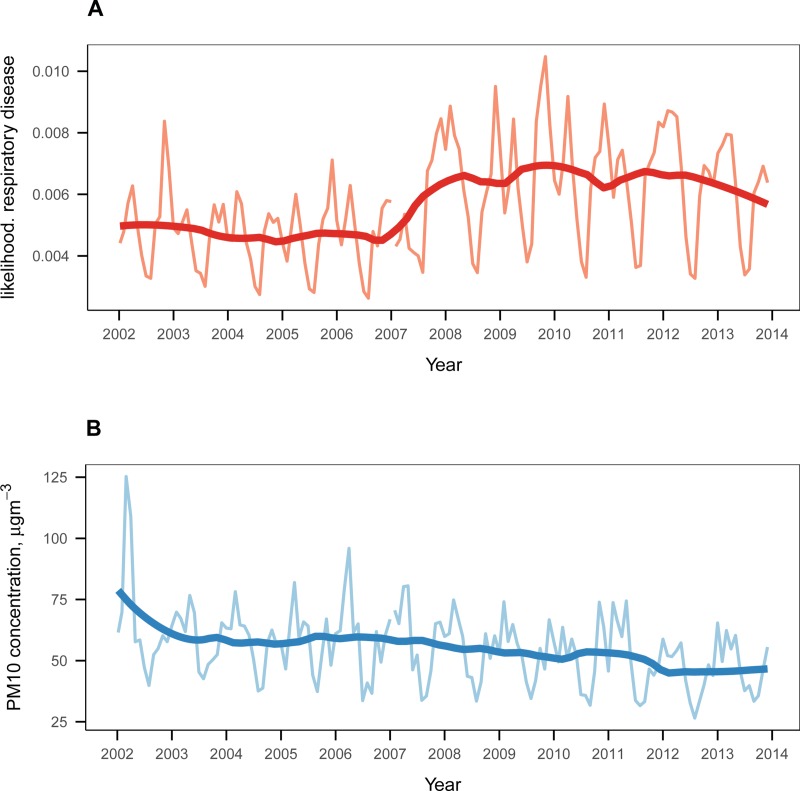
Time evolutions of the likelihood of respiratory disease and the PM_10_ concentration during 2002–2013. (**A**) The monthly time series of the average likelihood of respiratory disease are represented by the pink line, accompanied by a smoothed fit of the mean likelihood according to a generalized additive model (red line). (**B**) The same as (**A**) but the mean of the PM_10_ concentrations (light blue for the un-smoothed and blue for the smoothed series) are displayed.

The time trend of the likelihood of respiratory disease exhibits an abrupt increase in 2007 during the period of 2002–2013. In Fig 1A, the indicators of a doctor’s visit are aggregated to create the monthly mean time series of likelihood of respiratory disease. It shows no significant time trend between 2002 and 2006, but abruptly increases in 2007 and has maintained its overall level since then. This jump is due to a major healthcare reform in South Korea that lowered the patient’s share of medical expenses, which resulted in more frequent visits to hospitals.

The time trend of the monthly mean PM_10_ concentration shows a gradually decreasing trend between 2002 and 2013, possibly due to stricter enforcement of air quality controls (Fig 1B).

**Fig 2:**
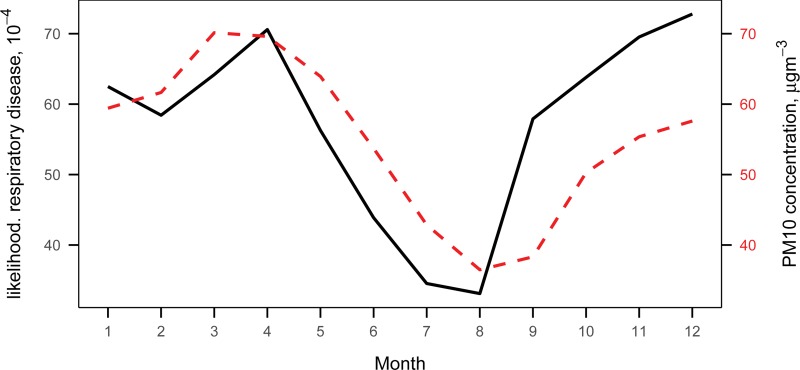
Monthly averages of the likelihood of respiratory disease and the PM_10_ concentration. The averages of the likelihood of respiratory disease by month (multiplied by 10^4^) are represented in black line (values are denoted on vertical axis on the left). The averages of the PM_10_ concentration by month are displayed in red dotted lines (values are denoted on vertical axis on the right).

The temporal relationship between the pollution and respiratory disease is clear on monthly means. Fig 2 shows the monthly means of the likelihood of respiratory disease and the PM_10_ concentration co-move, and exhibit a clear seasonal cycle: the likelihood of respiratory disease is highest in winter and lowest in summer. The monthly mean PM_10_ concentration follows a similar seasonal pattern: the concentration is highest during the winter and early spring, and lowest during the summer and early fall. This is due to the fact that more coal and other fossil fuels are burned during the colder seasons, and cold air near the ground, trapped by a layer of warm air due to temperature inversion, holds air pollutants, while warm air can rise easily and carry away pollutants during the warmer months [35].

### 2.2 Empirical strategy

Since the likelihood of respiratory disease was based on doctor’s visits, there can be a delay in time between exposure to air pollution and the actual visit. This temporal misalignment may be due to an individual’s delay in visiting a doctor, or a delay in the development of respiratory illness [36], which is related to different biological reactions to the particles [37]. The delay is different across types of symptoms, and typically lasts at most 6 days [38–41]. To incorporate possible effects of past exposure to pollution to doctors’ visits, we used the mean of the PM_10_ concentrations during the previous seven days, and called it the seven-day PM_10_ concentration.

In order to represent the difference in the underlying air quality of counties, we separated counties into the population-weighted quintiles according to their seven-day PM_10_ concentration such that (ii) the total population by quintile is comparable, and (ii) counties belonging to higher quintiles have higher seven-day PM_10_ concentration. The population of a county is defined as the mean of the actual population in the county, rather than the number of individuals in the sample, during the sample period. Since the seven-day PM_10_ concentration is supposed to represent the underlying air quality, counties that potentially have undergone large environmental changes are dropped from the analysis, i.e. the ones that have advanced to an urban city or become a hosting county for a major industrial complex during the sample period. S4 Table shows that the seven-day PM_10_ concentration was, by construction, lowest in the first quintile with 44.2 *μg*/*m*^3^ or microgram per cubic meter, and was highest in the fifth quintile with 63.6 *μg*/*m*^3^, while the mean is 54.8 *μg*/*m*^3^. The size of the population of a county is typically smaller among counties in the lower quintiles.

**Fig 3:**
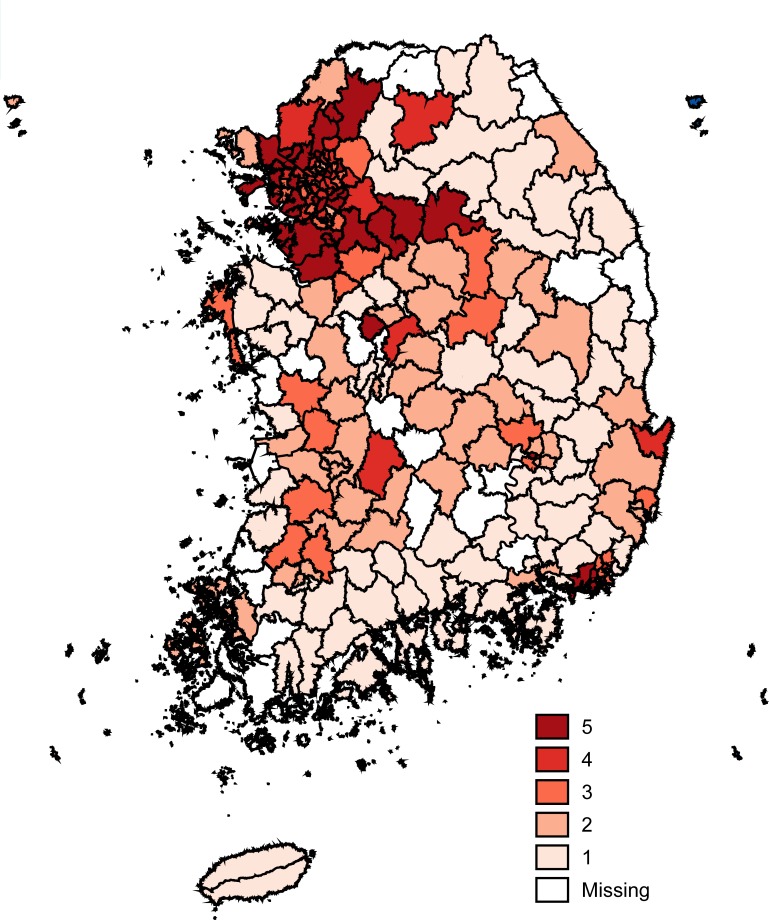
The choropleth maps of South Korea by county based on the seven-day PM_10_ concentration, and the mean likelihood of respiratory disease. The bordered areas of the map represent counties, and are shaded according to the quintile they belong to. The quintiles in **(A)** and **(B)** are created based on the seven-day PM_10_ concentration and mean likelihood of respiratory disease, respectively. The counties with no seven-day PM_10_ concentration observations or no individuals in the sample are labeled as missing.

Fig 3A shows the spatial distribution of the counties’ quintiles based on the seven-day PM_10_ concentration. Notice that the air quality in the neighboring counties seems to be correlated, especially in the large metropolitan area. For example, the capital city of South Korea, and its neighboring county in the upper left corner of Fig 3A mostly belong to the fifth quintile. Similarly, counties in the fifth quintile at the bottom right corner of the figure are the ones in the second largest city of the country. This spatial correlation may be due to the small size of the PM_10_ which makes it stay in the air for a long time, and can be scattered easily from a source to a neighboring region [42]. It may also be due to the similarity of economic activities and infrastructure among the neighboring counties.

On the other hand, the likelihood of respiratory disease is spatially mixed. To compare the distribution of seven-day PM_10_ concentration and the mean likelihood of respiratory disease, counties are separated into quintiles according to their mean likelihood of respiratory disease, such that the higher quintiles have a higher likelihood of respiratory disease. Fig 3B shows that compared to the distribution of the seven-day PM_10_ concentration, the likelihood of respiratory disease is more equally distributed across the country. It is also noticeable from Fig 3 that counties of high concentration are not necessarily associated with high probability of respiratory disease. According to our hypothesis, a part of this discrepancy is due to the behavioral adaptation by people to ambient air pollution.

If the cross sectional unit of analysis were a county, then the spatial regression would be an appropriate empirical strategy when it is suspected that the error terms are contemporaneously correlated, and so the likelihood of respiratory disease of neighboring counties are correlated. However, the unit of our analysis is a person, and no information about the exact location of people are available to compute the spatial correlation based on the Moran’s I [43]. Hence, the spatial regression is infeasible for our sample. Instead, we included the county dummies in the regression, as people from the same county face the identical degree of pollution. Furthermore, the heterogeneity among individuals is taken into account by including the individual dummies in the regression. Any remaining correlations in the error term will be removed by estimating the robust standard errors.

The key empirical strategy was to regress the daily indicator of a doctor’s visit with a resulting diagnosis of respiratory disease on the seven-day PM_10_ concentration across different quintiles by interacting the quintile dummies with the seven-day PM_10_ concentration. This specification is used to identify the potential difference in the impact of the seven-day PM_10_ concentration on the respiratory disease across counties in different quintiles. Our adaptation hypothesis would be supported if the estimated coefficients of the seven-day PM_10_ concentration in the regression were smaller in higher quintiles.

### 2.3 Regression model

The effect of the seven-day PM_10_ concentration on the likelihood of respiratory disease was estimated by the following the linear probability fixed effects regression:

$$R_{i,j,t}=R_{i,j,t-1}+\sum_{q=1}^{5}\gamma_{q}(P_{j,t}\times Z_{j,q})+T_{j,t}+D_{j\times m}+D_{y\times m}+H_{t}+c_{i}+\mu_{i,j,t}$$ (1)

Dependent variable *R*_*i*,*j*,*t*_ is a binary indicator that is equal to 1 if person *i* who lives in county *j* made a doctor’s visit with a resulting diagnosis of respiratory disease at day *t* and 0 otherwise. *P*_*j*,*t*_ is the seven-day PM_10_ concentration in the county *j* at day *t*. In order to estimate the differential effect of PM_10_ concentration across quintiles, *P*_*j*,*t*_ is multiplied by *Z*_*j*,*q*_ where *Z*_*j*,*q*_, *q* = 1,2,…,5, is an indicator variable that is equal to 1 if county *j* belongs to quintile *q* and 0 if otherwise.

The rest of the independent variables included factors that may influence the occurrence of respiratory disease and doctor’s visits: *R*_*i*,*j*,*t*−1_ is a one-day lagged dependent variable that reflects dynamics of change in the likelihood of respiratory disease. A dummy variable *T*_*j*,*t*_ represents a binary indicator of the comfortable temperature for outdoor activities and was equal to 1 if the daily maximum temperature was between 20°C and 26°C, and 0 if otherwise. *D*_*j*×*m*_ is a dummy for the interactions between county and month, which absorb differences in seasonal variations in respiratory patients across counties, so that the availability of hospitals and income differences will not confound the coefficient estimates. *D*_*y*×*m*_ is a dummy for interactions between year and month, which control factors that are common across counties, such as changes in national health insurance (the one in year 2007 in Fig 1A) and the environmental policy. *H*_*t*_ is a dummy equal to 1 if day *t* is a holiday or weekend, and 0 if otherwise. This holiday dummy captures the reality that many hospitals are closed during holidays and weekends, which restricts patients from visiting a doctor. The individual dummy *c*_*i*_ is included to control for the individual heterogeneity, such as the allergic predisposition and propensity for respiratory disease which can influence the likelihood of respiratory disease [44]. The term *μ*_*i*,*j*,*t*_ is a random error term. Finally, the estimated coefficients of *γ*_*q*_ in specification (1) represent the mean of within-individual variation of the seven-day PM_10_ concentration’s impact on the likelihood of respiratory disease, controlling for various confounding factors.

We estimated the regression specified in (1) using a linear likelihood model with fixed effects. However, since the dependent variable is binary, a nonlinear model such as a logistic regression may be more appropriate if it fits the data better than a linear model. However, a nonlinear model demands great computational resources to achieve the convergence of an iterative process of maximum likelihood when the sample size is large, with a large number of variables used in the estimation. Therefore, we restrict our analysis to the linear likelihood model.

## 3. Results

**Fig 4:**
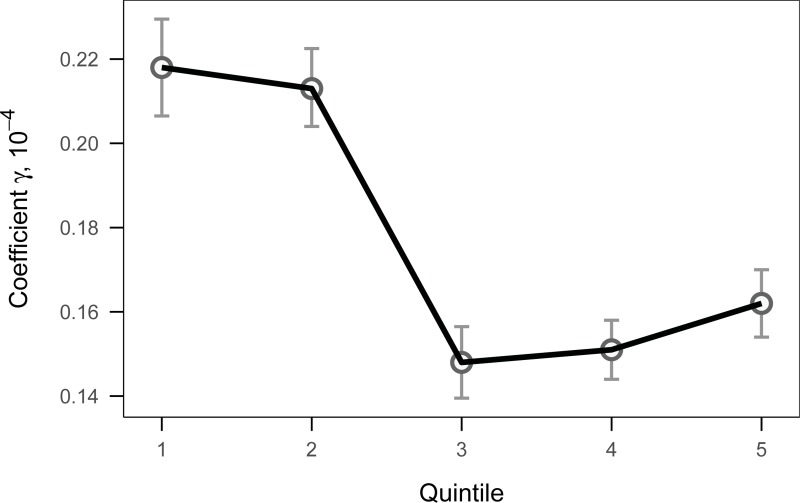
The regression estimates of the seven-day PM_10_ concentration by quintile. The estimated coefficients of the seven-day concentration of PM_10_ on the likelihood of respiratory disease using the whole sample are shown with the 95% error bars for each quintile. The errors are based on the robust standard errors, clustered by individual.

The estimated effects of the seven-day PM_10_ concentration on the likelihood of respiratory disease supported both the behavioral adaptation hypothesis and its limitations. Fig 4 shows that the first two quintiles have the higher coefficients of the seven-day PM_10_ concentration than the last three quintiles. A 1-standard-devivation increase in the seven-day PM_10_ concentration in the first two quintiles was associated with an increase of 0.054 percentage point (PP) in the likelihood of respiratory disease. The size of this effect is not trivial as it amounts to about 10% of the mean likelihood of respiratory disease in the whole sample. Moreover, these coefficients were statistically distinguishable from the coefficients for the higher quintiles, as the 95% confidence interval of estimated coefficient in the first two quintiles did not overlap with the others. This result implies that people who lived under greater air pollution (those in high quintiles) seem to adapt to their environment to suffer less from respiratory illness, compared to people who lived with cleaner air (those in lower quintiles).

Notice, however, that the effect of a 1-standard-deviation increase in the seven-day PM_10_ concentration gradually increased from 0.037 PP in the third quintile to 0.040 PP in the fifth quintile. This result can be interpreted as the limitation of behavioral adaptation: if the PM_10_ concentration was sufficiently high, the efficiency of adaptation was limited such that a greater concentration would lead to a higher chance of respiratory disease.

**Fig 5:**
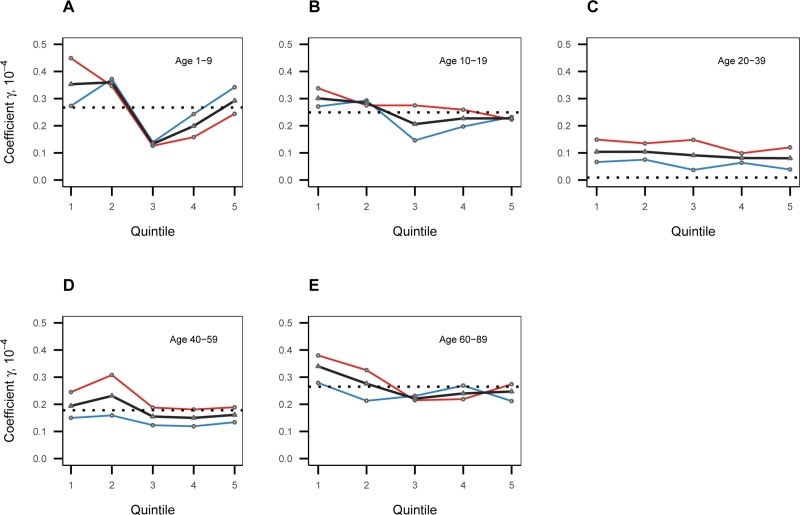
The regression estimates of the seven-day PM_10_ concentration by quintile, separately by age group and gender. For each age group, the coefficients of the seven-day PM_10_ concentration are estimated for all people (black line), and separately by gender (blue line for male and red line for female). The dotted horizontal lines denote the mean of the estimated coefficients based on all genders over quintiles.

To examine whether the estimated coefficients of the seven-day PM_10_ concentration are heterogeneous across the population, we separately estimated the regression in (1) by age group. The five age groups were: ages 1–9, 10–19, 20–39, 40–59, and 60–89. The regression results by age group are summarized in Fig 5.

Fig 5 reveals that there was a clear disparity by age group in the magnitude of the estimated coefficients, suggesting a difference in vulnerability to pollution by age. Specifically, the estimated coefficient for the young (ages 1–9 and 10–19) and the oldest (ages 60–89) is around 0.26×10^−4^, while it is only about 0.09×10^−4^ and 0.18×10^−4^ for people of ages 20–39 and 40–59, respectively (as shown by the dotted lines in Fig 5). This difference by age group is explained by the findings from previous studies that young people are more susceptible to the adverse effects of pollution, due to their immature immune systems, continuing development of their lungs during the early post-neonatal period [45–47], more frequent outdoor activities [48], and the elderly have weak respiratory functionality [49]. In other words, these factors could limit the efficiency of behavioral adaptation.

It is noticeable that the estimated coefficient for the people of age 1–9 increased the most from the third to the fifth quintile (Fig 5A). Recall that the estimated coefficient based on the whole population (Fig 4) also increased from the third quintile, which was related to the limitation to adaptation. Hence, the limitation may be due to the age-specific difference in degrees of vulnerability to pollution, and so is evident among the most vulnerable population of the youngest (aged 1–9).

We further investigated the possible differences by gender in the behavioral adaptation. Fig 5 reports the estimated coefficients of the seven-day PM_10_ concentration by gender for each age group. It shows that females generally suffer more from respiratory disease due to an increase in the seven-day PM_10_ concentration. Regarding behavioral adaptation, higher coefficients are generally associated with lower quintiles among females than among males, which suggests that females are more concerned with behavioral adaptation so that females in higher pollution regions suffer less from respiratory disease due to an increase in the PM_10_ concentration.

The limitation of the behavioral adaptation also differs by gender. Fig 5 shows a consistent increase in the coefficient of the seven-day PM_10_ concentration from the third to the fifth quintile among young males (age 1–9 and 10–19). In contrast, such an increase is only visible, with a smaller magnitude, among the youngest population (age 1–9) of females. This difference can be explained by the gender difference in lung development: Males have less mature lungs and narrower airways during childhood, which could make them more vulnerable to air pollution [50, 51]. However, the verdict on the gender difference in the relationship between air pollution and respiratory disease is far from unanimous in the literature [52–54]. Hence, further research is required to uncover the different mechanisms by which females and males respond to air pollution.

## 4. Discussion

In this paper, we did not explicitly measure the impact of specific behavioral adaptations. The weakness of this approach is that we could not attribute heterogeneity across quintiles to a specific adaptation measure. Instead, we claimed that the estimation results are consistent with predictions based on the behavioral adaptation hypothesis.

**Fig 6:**
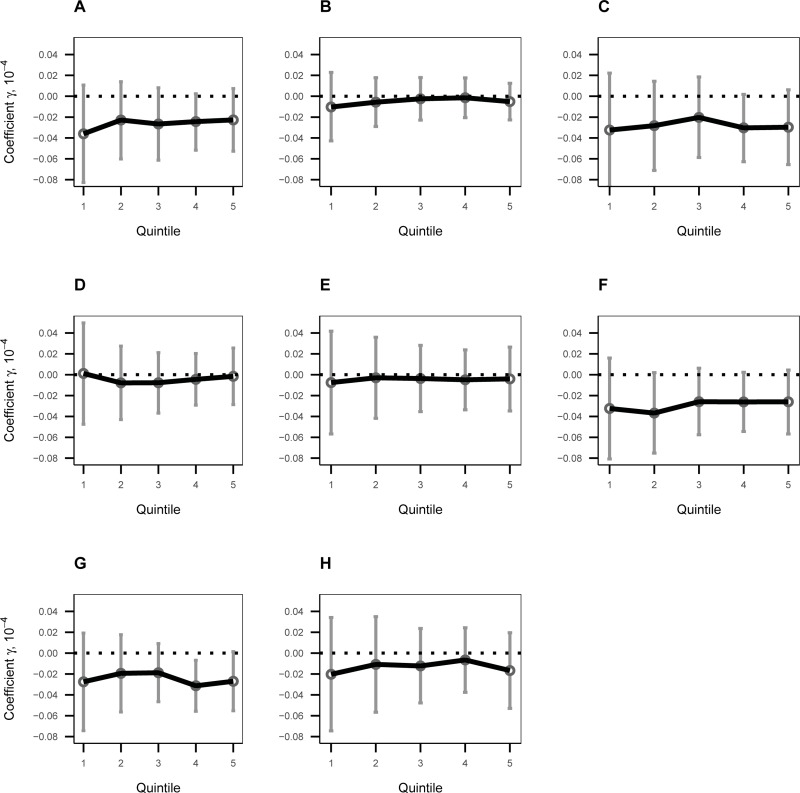
The estimated coefficients of seven-day PM_10_ concentration on non-respiratory patients by quintile. The estimated coefficients (multiplied by 10^4^) of the seven-day concentration on the likelihood of non-respiratory disease, listed in S5 Table, are presented with 95% error bars for each quintile: (**A**) Infectious and parasitic disease; (**B**) Nutritional and metabolic diseases; (**C**) Diseases of the eye and adnexa; (**D**) Diseases of the circulatory system; (**E**) Diseases of the digestive system; (**F**) Diseases of the skin and subcutaneous tissue; (**G**) Diseases of the musculoskeletal system and connective tissue; and (**H**) Injury, poisoning, and certain consequences of external causes.

However, we can validate our empirical specification that a difference in mean pollution concentration is a likely cause of a difference in daily changes in the likelihood of respiratory disease. For this purpose, we performed a “false experiment,” where we took advantage of data on patients diagnosed with non-respiratory diseases that were unlikely to be caused by exposure to ambient pollution. These diseases included burns/injuries and digestive diseases (see S5 Table for a complete list of these diseases). The goal of the experiment is to confirm that only the occurrence of respiratory illness was influenced by changes in the seven-day PM_10_ concentration. For the experiment, we estimated the same regression as in specification (1), but the dependent variable in the regression was replaced by a binary indicator of a doctor’s visit with a resulting diagnosis of non-respiratory disease. Fig 6 reports the estimated coefficients by quintile for non-respiratory disease. It showed that most of the estimated effects of the seven-day PM_10_ concentration on the likelihood of non-respiratory disease were statistically insignificant and did not exhibit systematic patterns across quintiles. These results suggested that the estimated difference in the effect of PM_10_ concentration on respiratory illness across quintiles was likely to be caused by a difference in the seven-day PM_10_ concentration across quintiles.

Another weakness of our estimation is that the estimated effect of the seven-day PM_10_ concentration ignored any long-term consequences (e.g. costs from complications and hospitalization) and indirect costs (e.g. loss in labor income) associated with the disease. The further research on long-term consequences associated with the development of respiratory disease due to increase in the PM_10_ concentration is required with more detailed information.

## 5. Conclusions

We examined the relationship between air pollution and the occurrence of respiratory disease using multiple dimensions of patient and pollution data. We found that people living in counties with lower mean pollution suffered more from respiratory disease due to an increase in ambient pollution. The result is consistent with the behavioral adaptation hypothesis. However, our results also indicate that when the mean pollution was beyond a critical level, behavioral adaptations seemed to be less efficient. This limitation was most severe among people who were most vulnerable to ambient pollution.

From the perspective of policy design, our results highlight the significance of behavioral adaptation in determining the actual impact of pollution in cases of respiratory disease. According to our analysis, counties in low quintiles or having low pollution were typically smaller in population, and thus were traditionally not at the center of environmental and health policy. However, according to our results, people living in these counties may suffer the most when quality of air depreciated quickly before they could adapt to it. Therefore, in contrast to conventional wisdom, preventative public health policy against air pollution should also be directed to population in these regions, as much as to people in high pollution regions. More effective policy design must consider both behavioral adaptation to air pollution, as well as its limitations.

## Supporting information

S1 Table A list of categories of respiratory disease by its frequency. (DOCX)

S2 Table The sample size by year and age group. (DOCX)

S3 Table Summary statistics. (DOCX)

S4 Table Summary statistics by quintile. (DOCX)

S5 Table Description of non-respiratory diseases categories. (DOCX)
