# Supplementary material for: Air pollution, respiratory illness and behavioral adaptation: Evidence from South Korea
Source: PLoS One. 2019 Aug 13;14(8):e0221098. doi: 10.1371/journal.pone.0221098 (PMC6692036; doi:10.1371/journal.pone.0221098)
Supplement: S1 Table — (DOCX) [file pone.0221098.s001.docx]

S1 Table. A list of categories of respiratory disease by its frequency

| KCD-6 | Descriptions | # of Diagnosis | Share |
| --- | --- | --- | --- |
| J00 – J06 | Acute upper respiratory infections | 296,239 | 47.40 |
| J20 – J22 | Other acute lower respiratory infections | 134,539 | 21.53 |
| J30 – J39 | Other diseases of upper respiratory tract | 117,592 | 18.82 |
| J40 – J47 | Chronic lower respiratory diseases | 54,128 | 8.66 |
| J09 – J18 | Influenza and pneumonia | 14,222 | 2.28 |
| R06–R09 | Symptoms involving the respiratory systems | 6,774 | 1.08 |
| J95 – J99 | Other diseases of the respiratory system | 584 | 0.09 |
| J90 – J94 | Other diseases of pleura | 490 | 0.08 |
| J80 – J84 | Other respiratory diseases principally affecting the interstitium | 182 | 0.03 |
| J85 – J86 | Suppurative and necrotic conditions of lower respiratory tract | 106 | 0.02 |
| J60 – J70 | Lung diseases due to external agents | 97 | 0.02 |
