# Supplementary material for: Air pollution, respiratory illness and behavioral adaptation: Evidence from South Korea
Source: PLoS One. 2019 Aug 13;14(8):e0221098. doi: 10.1371/journal.pone.0221098 (PMC6692036; doi:10.1371/journal.pone.0221098)
Supplement: S2 Table — (DOCX) [file pone.0221098.s002.docx]

S2 Table. The sample size by year and age group

| Year | All | Men  (fraction) | Ages | | | | |
| --- | --- | --- | --- | --- | --- | --- | --- |
|  |  |  | 1-9 | 10-19 | 20-39 | 40-59 | 60-89 |
| All years (mean) | 25,747 | 0.498 | 1,375 | 3,661 | 8,696 | 8,135 | 3,878 |
| 2002 | 23,093 | 0.501 | 3,065 | 3,198 | 8,375 | 5,962 | 2,493 |
| 2003 | 24,215 | 0.501 | 2,858 | 3,307 | 8,705 | 6,471 | 2,874 |
| 2004 | 24,747 | 0.501 | 2,545 | 3,440 | 8,787 | 6,884 | 3,091 |
| 2005 | 25,749 | 0.499 | 2,281 | 3,695 | 8,962 | 7,487 | 3,324 |
| 2006 | 25,471 | 0.498 | 1,910 | 3,752 | 8,753 | 7,614 | 3,442 |
| 2007 | 26,873 | 0.498 | 1,627 | 3,943 | 9,109 | 8,243 | 3,951 |
| 2008 | 26,543 | 0.499 | 1,255 | 3,938 | 8,903 | 8,379 | 4,068 |
| 2009 | 26,311 | 0.499 | 917 | 3,880 | 8,692 | 8,594 | 4,228 |
| 2010 | 26,274 | 0.498 | 526 | 3,937 | 8,583 | 8,898 | 4,330 |
| 2011 | 26,312 | 0.498 | 244 | 3,809 | 8,556 | 9,225 | 4,478 |
| 2012 | 26,266 | 0.498 | 0 | 3,648 | 8,498 | 9,389 | 4,731 |
| 2013 | 26,277 | 0.497 | 0 | 3,243 | 8,396 | 9,646 | 4,992 |
