# Supplementary material for: Air pollution, respiratory illness and behavioral adaptation: Evidence from South Korea
Source: PLoS One. 2019 Aug 13;14(8):e0221098. doi: 10.1371/journal.pone.0221098 (PMC6692036; doi:10.1371/journal.pone.0221098)
Supplement: S3 Table — (DOCX) [file pone.0221098.s003.docx]

S3 Table. Summary statistics

|  | Mean | Standard deviation |
| --- | --- | --- |
| PM_10_ concentration $(\mu g/m^{3})$ | 54.881 | 37.466 |
| Likelihood of respiratory disease |  |  |
| All | 0.0057 | 0.075 |
| Ages 1-9 | 0.0162 | 0.126 |
| 10-19 | 0.0063 | 0.079 |
| 20-39 | 0.0042 | 0.065 |
| 40-59 | 0.0047 | 0.069 |
| 60-89 | 0.0066 | 0.081 |
| Gender Males | 0.0049 | 0.070 |
| Females | 0.0064 | 0.080 |
| Holiday | 0.1742 | 0.379 |
| Comfortable temperature | 0.1949 | 0.396 |
