# Supplementary material for: Air pollution, respiratory illness and behavioral adaptation: Evidence from South Korea
Source: PLoS One. 2019 Aug 13;14(8):e0221098. doi: 10.1371/journal.pone.0221098 (PMC6692036; doi:10.1371/journal.pone.0221098)
Supplement: S4 Table — (DOCX) [file pone.0221098.s004.docx]

S4 Table. Summary statistics by quintile.

| Variable | Quintile | | | | |
| --- | --- | --- | --- | --- | --- |
|  | 1 | 2 | 3 | 4 | 5 |
| The seven-day PM_10_ concentration | 44.2 | 50.4 | 54.8 | 58.2 | 63.6 |
| Likelihood of respiratory disease | 0.0058 | 0.0057 | 0.0058 | 0.0055 | 0.0059 |
| Population | 128,335 | 187,725 | 301,290 | 327,713 | 261,164 |
| Number of counties | 77 | 55 | 34 | 31 | 39 |
