# Supplementary material for: Air pollution, respiratory illness and behavioral adaptation: Evidence from South Korea
Source: PLoS One. 2019 Aug 13;14(8):e0221098. doi: 10.1371/journal.pone.0221098 (PMC6692036; doi:10.1371/journal.pone.0221098)
Supplement: S5 Table — (DOCX) [file pone.0221098.s005.docx]

S5 Table. Description of non-respiratory diseases categories

| KDC-6 | Descriptions | Examples |
| --- | --- | --- |
| B | Infectious and parasitic disease | Hepatitis, HIV |
| E | Nutritional and metabolic diseases | Diabetes, Malnutrition |
| H | Diseases of the eye and adnexa | Glaucoma, Otitis media |
| I | Diseases of the circulatory system | Cerebrovascular and Rheumatic heart diseases |
| K | Diseases of the digestive system | Endocrine, Nutritional, and Metabolic diseases |
| L | Diseases of the skin and subcutaneous tissue | Dermatitis, Eczema |
| M | Diseases of the musculoskeletal system and connective tissue | Arthropathies, Osteopathies |
| S | Injury, poisoning, and certain consequences of external causes | Frostbite, Burns, Corrosions |

Column 1 shows the first letter of disease codes according to the Korea Classification of Disease (KCD-6). Columns 2 and 3 present short descriptions of the diseases and examples, respectively.
